# A Biochemical Approach to Detect Oxidative Stress in Infertile Women Undergoing Assisted Reproductive Technology Procedures

**DOI:** 10.3390/ijms19020592

**Published:** 2018-02-16

**Authors:** Matteo Becatti, Rossella Fucci, Amanda Mannucci, Victoria Barygina, Marco Mugnaini, Luciana Criscuoli, Claudia Giachini, Francesco Bertocci, Rita Picone, Giacomo Emmi, Paolo Evangelisti, Francesca Rizzello, Cinzia Cozzi, Niccolò Taddei, Claudia Fiorillo, Maria Elisabetta Coccia

**Affiliations:** 1 Department of Experimental and Clinical Biomedical Sciences “Mario Serio”, University of Florence, Viale Morgagni 50, 50134 Florence, Italy; matteo.becatti@unifi.it (M.B.); rossella.2f@gmail.com (R.F.); amanda.mannucci@unifi.it (A.M.); v.barygina@gmail.com (V.B.); criscuolil@aou-careggi.toscana.it (L.C.); claudia.giachini@unifi.it (C.G.); niccolo.taddei@unifi.it (N.T.); mariaelisabetta.coccia@unifi.it (M.E.C.); 2 Department of Information Engineering and Mathematics, University of Siena, 53100 Siena, Italy; mugnaini@dii.unisi.it; 3 Assisted Reproduction Center, Careggi University Hospital, 50134 Florence, Italy; francesco.bertocci61@gmail.com (F.B.); ritapicone87@gmail.com (R.P.); evangelistip@aou-careggi.toscana.it (P.E.); francesca.rizzello@gmail.com (F.R.); cozzici@aou-careggi.toscana.it (C.C.); 4 Department of Experimental and Clinical Medicine, University of Florence, 50134 Florence, Italy; giacomo.emmi@unifi.it

**Keywords:** oxidative stress, in vitro fertilization (IVF), infertile women, follicular fluid (FF), granulosa cells

## Abstract

Oxidative stress plays a major role in critical biological processes in human reproduction. However, a reliable and biologically accurate indicator of this condition does not yet exist. On these bases, the aim of this study was to assess and compare the blood and follicular fluid (FF) redox status of 45 infertile subjects (and 45 age-matched controls) undergoing in vitro fertilization (IVF), and explore possible relationships between the assessed redox parameters and IVF outcomes. Reactive Oxygen Species (ROS) production, assessed by flow cytometry analysis in blood leukocytes and granulosa cells, significantly increased (*p* < 0.05) in infertile patients. Also, oxidative stress markers—ThioBarbituric Acid-Reactive Substances (TBARS) as an index of lipid peroxidation, and Oxygen Radical Absorbance Capacity (ORAC) to account for total antioxidant capacity, both assayed by fluorometric procedures—in blood and FF were significantly (*p* < 0.001) modified in infertile patients compared to the control group. Moreover, a significant correlation between blood redox markers and FF redox markers was evident. An ORAC/TBARS ratio, defined as the redox index (RI), was obtained in the plasma and FF of the patients and controls. In the patients, the plasma RI was about 3.4-fold (*p* < 0.0001) lower than the control, and the FF RI was about six-fold (*p* < 0.0001) lower than the control. Interestingly, both the plasma RI and FF RI results were significantly correlated (*p* < 0.05) to the considered outcome parameters (metaphase II, fertilization rate, and ongoing pregnancies). Given the reported findings, a strict monitoring of redox parameters in assisted reproductive techniques and infertility management is recommended.

## 1. Introduction

Oxidative stress is a condition caused by an imbalance between reactive oxygen or nitrogen species (ROS/RNS) production, and/or a decrease in antioxidant defense systems. This process is initially responsible for an adaptive response consisting of the induction of an antioxidant response, and following antioxidant depletion, cellular injury and dysfunction [1,2,3]. The loss of balance between oxidant and antioxidant molecules in serum and in follicular fluid (FF) has been suggested to be responsible for anomalous oocyte development, due to DNA and cell membrane damage, which would then result in reduced egg quality, but also in altered fertilization, embryo quality, implantation, and embryonic development [4,5]. Several studies have reported signs of oxidative stress in serum and in the FF of infertile women [6,7,8], but data on the simultaneous presence of oxidative stress markers in blood (particularly plasma and leukocytes), FF, and granulosa cells are lacking. Such information would be useful for a better understanding of the ethiopathogenic mechanisms involved in infertility, due to the still debated role of oxidative stress in infertile patients. Interestingly, FF composition reflects all of the metabolic and hormonal processes occurring in the microenvironment of the maturing oocyte, and can represent a predictor of outcome parameters such as fertilization, embryo cleavage, and pregnancy rates in IVF [9]. At present, in the literature, contrasting results related to the detrimental or beneficial effects of ROS during in vitro fertilization (IVF) procedures are present. Some authors suggested that oxidative stress alters the oocyte and embryo quality, and thus the fertilization rate [10], while other authors [11] reported that signs of lipid peroxidation in FF do not reflect the reproductive potential of oocytes. Furthermore, it has been shown that higher ROS levels and signs of lipid peroxidation [12,13] are present in women who became pregnant by IVF, but also that a higher total antioxidant capacity (TAC) is associated with increased fertilization potential in women undergoing IVF [7]. Thus, there does not yet exist a general consensus on the role of oxidative stress in the processes regulating fertilization and embryonic development, nor on the relationship between the redox parameters in blood and FF, and their effects on oocyte, embryo quality, and finally IVF outcomes.

Based on this background, in this study, we estimated both in infertile and control patients through: (1) leukocyte ROS production and plasma oxidative stress markers in blood samples; and (2) granulosa cell ROS production and oxidative stress markers in FF samples. Indeed, our primary aim was to compare the obtained redox index (RI) in plasma and in the follicular fluid of infertile patients, and search for a correlation between these parameters and the outcomes of IVF procedures.

## 2. Results

### 2.1. Assessment of Oxidative Stress in Blood Samples and in Granulosa Cells

**Figure 1:**
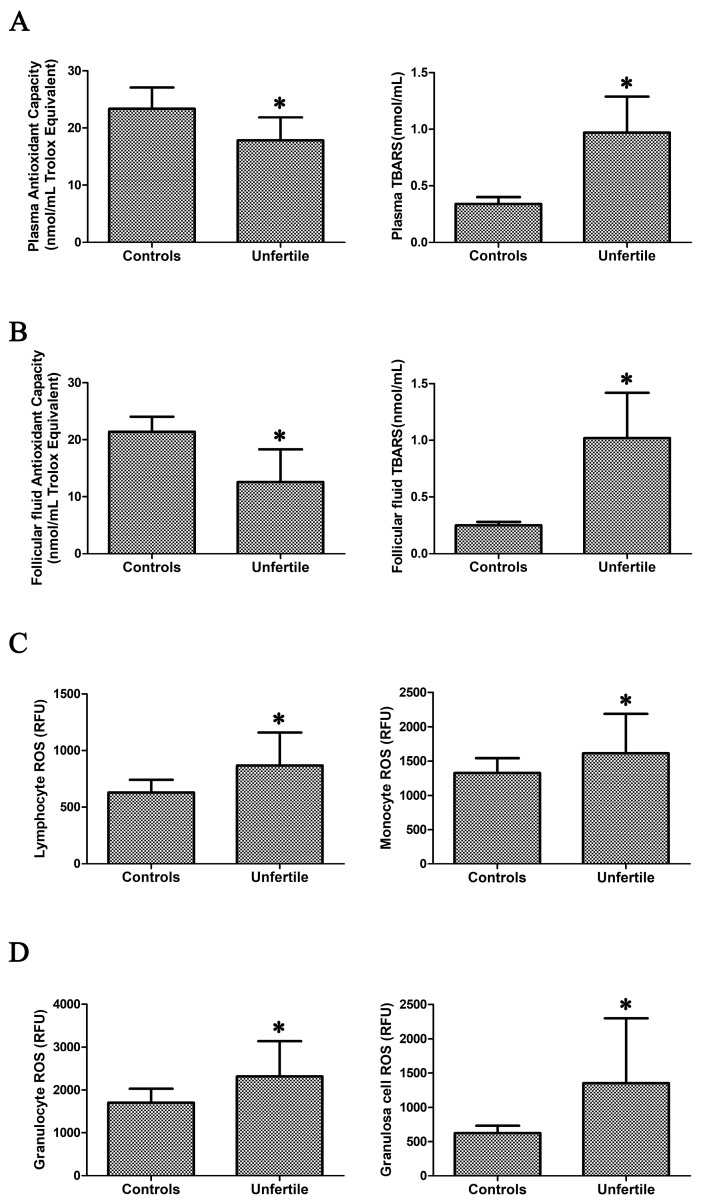
Oxidative stress parameters in the plasma and follicular fluid of infertile patients and controls. Total antioxidant capacity and lipid peroxidation in plasma (**A**) and follicular fluid (**B**), leukocyte and granulosa cells reactive oxygen species (ROS) production (**C**,**D**) in infertile patients (*n* = 45) and controls (*n *= 45). * indicates that differences are statistically significant at the *p* < 0.05 level.

Oxidative stress in the plasma of infertile patients was indicated (Figure 1A) by the significantly decreased total antioxidant capacity (23.42 ± 3.69 vs. 17.86 ± 4.02, *p* < 0.0001) and the significantly increased thiobarbituric acid-reactive substances (TBARS) level (an index of lipid peroxidation, 0.34 ± 0.06 vs. 0.97 ± 0.32, *p* < 0.0001).

For plasma total antioxidant capacity and lipid peroxidation, we calculated a sample size (to reach statistical significance, *p* < 0.05, between groups) of 10 and 19, respectively. In infertile patients, redox alterations were also evident in FF (Figure 1B), where total antioxidant capacity was significantly reduced (21.35 ± 2.72 vs. 12.59 ± 5.72, *p* < 0.0001), and lipid peroxidation was significantly increased (0.25 ± 0.03 vs. 1.02 ± 0.40, *p* < 0.0001). For FF total antioxidant capacity and lipid peroxidation, we calculated a sample size (to reach statistical significance, *p* < 0.05, between groups) of 13 and 20, respectively.

The presence of oxidative stress in infertile patients was also confirmed by the significantly enhanced ROS production in leukocytes (lymphocyte, monocyte, and granulocyte) and granulosa cells (Figure 1C,D) compared to controls. We calculated a sample size (to reach statistical significance, *p* < 0.05, between groups) of 13, 28, and 29, for lymphocytes, monocytes, and granulocytes, and of 20 for granulosa cells, respectively.

### 2.2. Redox Index Calculated as ORAC/TBARS Ratio as a Mirror of Oxidative Stress

**Figure 2:**
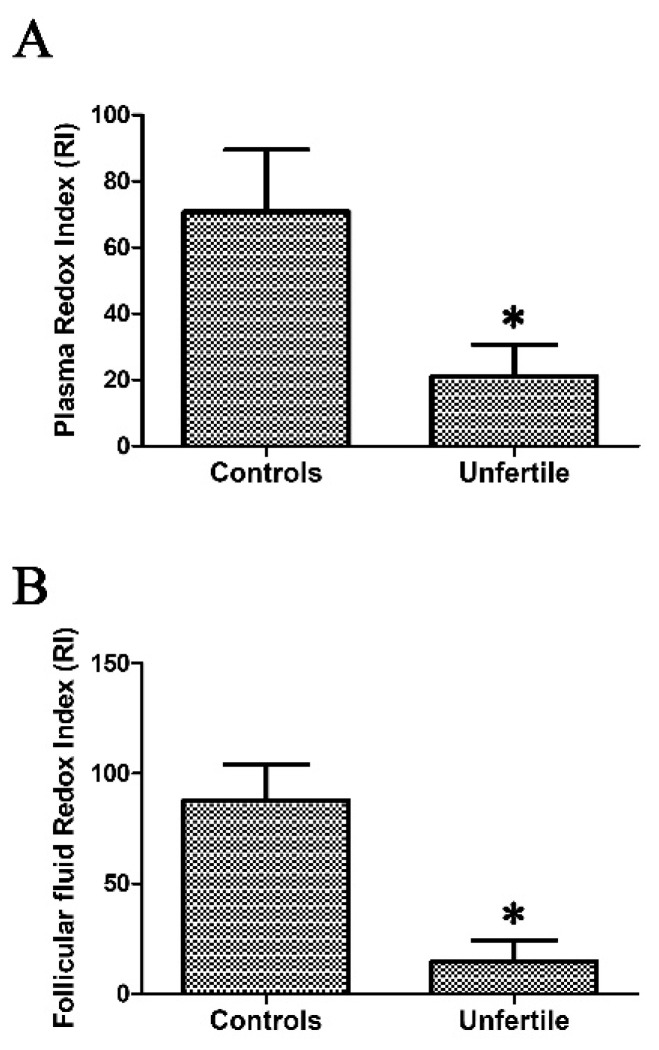
(**A**) Plasma redox index (RI) and (**B**) follicular fluid redox index values in infertile patients (*n* = 45) and controls (*n* = 45). * indicates that differences are statistically significant at the *p* < 0.05 level.

To achieve a reliable estimation of the redox status in infertile patients and controls, we calculated, both in plasma and in FF, a redox index (RI) obtained as an oxygen radical absorbance capacity (ORAC)/TBARS ratio. In infertile patients, the plasma RI result was about 3.4-fold (*p* < 0.0001) lower than in controls, and the FF was six-fold (*p* < 0.0001) lower than in controls (Figure 2). For the plasma RI and FF RI, we calculated a sample size (to reach statistical significance, *p* < 0.05, between groups) of 16 and 9, respectively.

### 2.3. Correlation between Investigated Parameters

**Figure 3:**
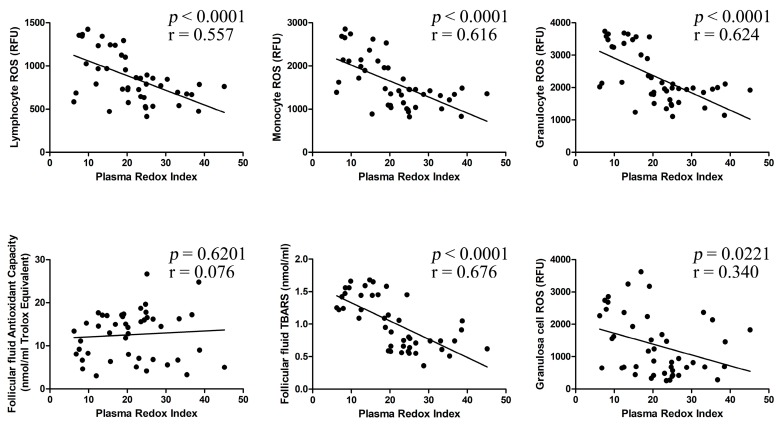
Plasma redox index correlation analyses. Relationship among the investigated redox parameters and the plasma redox index. Statistical significance was considered at the *p* < 0.05 level.

As shown in Figure 3, plasma RI significantly correlates with lymphocyte ROS (*r* = 0.557, *p* < 0.0001), monocyte ROS (*r* = 0.616, *p* < 0.0001), granulocyte ROS (*r* = 0.624, *p* < 0.0001), FF TBARS levels (*r* = 0.676, *p* < 0.0001), and granulosa cell ROS (*r* = 0.340, *p* < 0.05), but not with FF antioxidant capacity (*r* = 0.076, *p* = 0.6201).

**Figure 4:**
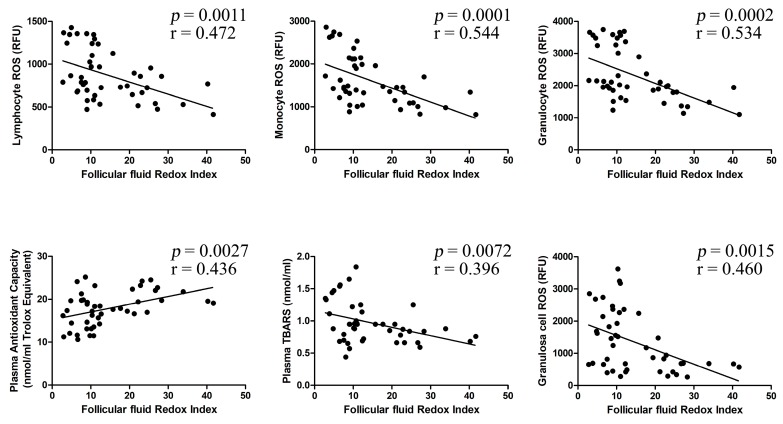
Follicular fluid redox index correlation analyses. Relationship among the investigated redox parameters and the follicular fluid redox index values. All of the correlation analyses were statistically significant at the *p* < 0.05 level.

Figure 4 shows that FF RI significantly correlates with lymphocyte ROS (*r* = 0.472, *p* < 0.005), monocyte ROS (*r* = 0.544, *p* < 0.0001), granulocyte ROS (*r* = 0.534, *p* < 0.001), plasma antioxidant capacity (*r* = 0.436, *p* < 0.005), plasma TBARS (*r* = 0.396, *p* < 0.01), and granulosa cell ROS (*r* = 0.460, *p* < 0.005).

At partial correlation analyses, oxidative stress-related parameters (ROS production and lipid peroxidation) were significantly related with plasma RI and FF RI after controlling for endometriosis, age, smoking habits, body mass index (BMI), in vitro fertilization-embryo transfer (IVF-ET)/intracytoplasmic sperm injection (ICSI), follicle stimulating hormone (FSH), luteinizing hormone (LH), estradiol (E2), Anti-Müllerian Hormone (AMH) and duration of infertility (Table 1). In particular, plasma RI is significantly related to plasma ORAC (*r* = 0.838, *p *< 0.001), plasma TBARS (*r* = −0.905, *p *< 0.001), lymphocyte ROS (*r* = −0.640, *p *< 0.001), monocyte ROS (*r* = −0.669, *p *< 0.001), granulocyte ROS (*r* = −0.724, *p *< 0.001), FF TBARS (*r* = −0.748, *p *< 0.001), and granulosa cell ROS (*r* = −0.400, *p *= 0.019). FF RI is significantly related to plasma ORAC (*r* = 0.474, *p *= 0.005), plasma TBARS (*r* = −0.506, *p *= 0.002), lymphocyte ROS (*r* = −0.434, *p *= 0.011), monocyte ROS (*r* = −0.520, *p *= 0.002), granulocyte ROS (*r* = −0.489, *p *= 0.003), FF ORAC (*r* = 0.761, *p *< 0.001), FF TBARS (*r* = −0.520, *p *= 0.002), and granulosa cell ROS (*r* = −0.528, *p *= 0.001).

### 2.4. Systemic Oxidative Stress Parameters are Related to Assisted Reproductive Technologies (ART) Outcome

In order to verify the possible role of redox alterations in the main outcomes of ART procedures in infertile patients, we performed suitable correlation analyses among the plasma/FF oxidative stress-related parameters and fertilization rate (%), and retrieved the oocyte metaphase II (%) ongoing pregnancies indexes.

As shown in Table 2, at partial correlation analyses, % metaphase II oocytes significantly correlated with plasma TBARS (*r* = −0.540, *p *= 0.001) and ORAC (*r* = 0.524, *p *= 0.001), lymphocyte ROS (*r* = −0.542, *p *= 0.001), monocyte ROS (*r* = −0.494, *p *= 0.003), granulocyte ROS (*r* = −0.572, *p *< 0.001), granulosa cell ROS (*r* = −0.654, *p *< 0.001), FF TBARS (*r* = −0.451, *p *= 0.007), and both with plasma RI (*r* = 0.463, *p *= 0.006) and FF RI (*r* = 0.459, *p *= 0.006) values, but not with FF antioxidant capacity (*r* = −0.212, *p *= 0.229), after controlling for endometriosis, age, smoking habits, BMI, IVF-ET/ICSI, FSH, LH, estradiol, AMH, and duration of infertility. In addition, at partial correlation analyses, fertilization rate (%) significantly correlated with plasma TBARS (*r* = −0.685, *p *< 0.001), ORAC (*r* = 0.469, *p *= 0.005), monocyte ROS (*r* = −0.367, *p *= 0.033), FF TBARS (*r* = −0.476, *p *= 0.004), and with both plasma RI (*r* = 0.520, *p *= 0.002) and FF RI (*r* = 0.413, *p *= 0.015) values, but not with lymphocyte ROS (*r* = −0.312, *p *= 0.072), granulocyte ROS (*r* = −0.333, *p *= 0.054), granulosa cell ROS (*r* = −0.268, *p *= 0.126), or FF antioxidant capacity (*r* = 0.316, *p *= 0.069), also after controlling for endometriosis, age, smoking habits, BMI, IVF-ET/ICSI, FSH, LH, estradiol, AMH, and duration of infertility. 

Finally, at partial correlation analyses, ongoing pregnancies significantly correlated with plasma TBARS (*r* = −0.471; *p *= 0.005) and ORAC (*r* = 0.504; *p *= 0.002), lymphocyte ROS (*r* = −0.384; *p *= 0.025), monocyte ROS (*r* = −0.433; *p *= 0.010), granulocyte ROS (*r* = −0.465; *p *= 0.006), granulosa cell ROS (*r* = −0.357; *p *= 0.038), FF TBARS (*r* = −0.351; *p *= 0.042), FF antioxidant capacity (*r* = 0.404; *p *= 0.018), and both with plasma RI (*r* = 0.549; *p *= 0.001) and FF RI (*r* = 0.518; *p *= 0.002), also after controlling for endometriosis, age, smoking habits, BMI, IVF-ET/ICSI, FSH, LH, estradiol, AMH, and duration of infertility.

## 3. Discussion

Our data clearly demonstrate that in infertile women undergoing IVF, the blood redox status is significantly altered and reflects the FF redox status, thus giving valuable information on ovarian physiology and the oocyte environment/follicular microenvironment. Interestingly, both the plasma and FF RI values show significant correlations with the considered indicators of IVF outcomes, even after adjustment for several confounders.

Although a certain amount of ROS is required under physiological conditions, an altered balance between pro-oxidant and antioxidant molecules may have deleterious effects on folliculogenesis and adequate embryo development [14,15,16]. Indeed, ROS deriving from several sources (i.e., the electron leakage of the inner mitochondrial membrane during oxidative phosphorylation) can damage cellular macromolecules during follicle growth. Consequently, antioxidants represent important defense mechanisms that allow cells to function within an oxidative environment, including the transient rise in ROS activity that accompanies ovulation [17,18].

Oxidative stress, which displays a fundamental role in follicle development and oocyte maturation, has been studied by several strategies. In our study, markers of oxidative stress both in blood and in FF, and ROS production in leukocyte subpopulations from infertile patients were significantly increased compared to controls. Indeed, as we previously reported [19], peripheral leukocytes represent a reliable model for studying the pathophysiology of oxidative stress-mediated homeostasis variations, which can be responsible for cell dysfunction and cell injury. Leukocytes reflect the condition of the whole organism, and thus represent a valuable model to study systemic oxidative stress-related disorders [20]. Indeed, one of the main results of our study is the significant correlation among leukocyte ROS production both with plasma RI and FF RI, and granulosa cell ROS.

Interestingly, the results of the statistical analyses indicate that this correlation remained significant even after adjustment for endometriosis, age, smoking habits, BMI, IVF-ET/ICSI, FSH, LH, estradiol, AMH, and duration of infertility.

In reproductive functions, the follicular microenvironment plays a critical role in oocyte maturation by providing an adequate environment for oocyte growth. FF provides a good amount of information about the biochemical status of the follicles, because it contains several metabolites that are useful for oocyte development. Changes in FF composition might influence oocyte quality, and thus affect fertilization, early embryonic development, and subsequent pregnancy [21]. In particular, FF contains molecules whose cysteine residues may be involved in sensing and buffering the local redox conditions, which represents a critical issue in reproduction. In fact, in FF, several important proteins regulating follicle growth and oocyte quality exhibit cysteine residues at specific points, whose oxidation would result in functional loss. Therefore, the preservation of controlled redox conditions in the FF is essential for the fine-tuned oocyte maturation process [13,22]. In contrast, its disturbance enhances the susceptibility to the establishment of reproductive disorders that would require the intervention of reproductive medicine technology. In FF, ROS and antioxidants produced by granulosa cells, endothelial cells, and leukocytes are also present [23]. Although ROS are essential in some female reproductive functions, including the ovulatory response [24], in excess they might have a negative impact, especially on estradiol levels, which are an important predictor of ovarian response [25]. ROS might also damage steroidogenesis, and consequently oocyte maturation and ovulation [8,26]. Our results indicate that signs of oxidative stress (increased lipid peroxidation markers and reduced antioxidant capacity) are present in the FF of infertile patients, which is in line with other reports showing FF oxidative stress and its correlation with poor-oocyte/embryo quality and low fertilization rates [27,28,29]. Interestingly, the FF redox index was not only significantly correlated with plasma TBARS, ORAC, and plasma RI, it was also correlated with leukocyte and granulosa cell ROS production, even after adjustment for several confounders. High 8OHdG levels in the granulosa cells of infertile women undergoing IVF have been negatively correlated with fertilization rates and embryo quality [30]. Supporting these data, other authors [31] demonstrated that increased 8OHdG concentrations in the FF of women undergoing IVF are associated with oocyte degeneration, suggesting that oxidative stress in the follicular compartment has deleterious effects on the oocyte.

Our data are in agreement with a report showing lower vitamin E concentrations in the FF of women with infertility related to endometriosis [32], but are in contrast with another study reporting both increased 8OHdG and a higher vitamin E concentration in the FF of women with endometriosis. These data probably indicate an up-regulation of the follicular antioxidant system in an unsuccessful attempt to neutralize reactive species and prevent oxidative damage to oocytes [33].

Another important result emerging from our study is the increased ROS production in granulosa cells from infertile patients. Granulosa cells ensure the successful maturation and developmental competency of oocytes by providing nutrients and maturation-enabling factors. Moreover, they protect oocytes from oxidative stress injury through their own antioxidant system during the maturation of oocytes [34,35]. Granulosa cells are particularly sensitive to ROS, which play a key role in their apoptosis-induction [4,5,36,37], in particular to endogenous H_2_O_2_, which is an important signaling molecule. At the same time, when present at high levels, ROS may cause cell dysfunction and cell death. Accordingly, it has been shown that reduced glutathione depletion sensitizes granulosa cells to toxicant-induced apoptosis [38], but the mechanism underlying cell toxicity induced by ROS is less known. Among our main results, we found that granulosa cell ROS production was significantly correlated with leukocyte ROS production and FF RI. 

The redox index (RI) that we established in our study populations, both in plasma and in FF samples, was obtained from the ratio between antioxidants and oxidative stress markers (ORAC/TBARS). In our opinion, together with cellular (leukocytes and granulosa cells) ROS production estimation, the RI can give valuable information about global oxidative stress occurrences. In fact, the performed statistical analyses indicate a strict relationship between plasma RI and FF RI, and their significant correlation with the estimated blood/FF redox parameters. These correlations remained significant even after adjustment for endometriosis, age, smoking habits, BMI, IVF-ET/ICSI, FSH, LH, estradiol, AMH, and the duration of infertility, confirming that systemic oxidative stress reflects FF oxidative stress. In this regard, other authors [39] reported a lower antioxidant capacity in the peritoneal fluid of women with infertility (due to endometriosis) compared to controls, indicating that the serum compartment reflects the redox status of the peritoneal microenvironment.

Increased plasma and FF oxidative stress markers and decreased levels of antioxidants have been already related to poor in vitro fertilization (IVF) outcomes [40,41,42]. Indeed, several authors studied follicular development, oocyte quality, fertilization, embryonic development, and female infertility in relation to oxidative stress, both at systemic and local levels [43,44,45,46]. However, due to the different strategies (analysis of various biological samples) and methodologies (analysis of systemic levels of antioxidants versus gene expression) used in the different studies about the redox state in the ovary, results are often difficult to compare, and are occasionally contradictory. In addition, our findings reveal that the selected technique (IVF-ET or ICSI) is not associated with different ROS levels or outcome parameters. Data from our clinic also confirm that the fertilization rate is not significantly different when using IVF-ET or ICSI, and therefore, the use of the two different techniques does not represent a bias for the present study.

This study is not without limitations. First, the study population is small. However, strict eligibility criteria are necessary to increase the internal validity, eliminating other factors that are potentially related to oxidative stress and compromised oocyte quality. Second, whether the analysis of a single follicle is representative of the set of follicles that responded to ovarian stimulation cannot be explained. However, since a longer time of anesthesia and repeated ovarian punctures might promote follicular and/or systemic oxidative stress, in order to increase the internal validity of the present study, we chose to aspirate only the entire content of the first follicle of the first ovary punctured. Third, although our findings suggest plasma RI values and leukocyte ROS production as potential predictors of positive IVF outcomes, larger studies are needed for validation. Finally, we want to underline that peripheral blood collection is easier, more practical, less invasive, and less susceptible to sampling inadequacy than FF collection.

In conclusion, several studies performed so far indicate that oxidative stress negatively affects reproductive potential, and is associated with poor gamete quality and abnormally early embryo development. However, a clinically reliable, biologically accurate indicator of oxidative stress condition does not yet exist, and is still difficult to identify.

We think that the use of the parameters suggested here could help define the critical role of oxidative stress markers and their optimum levels in the female reproductive system, and improve the success rate of assisted reproductive techniques and infertility management.

## 4. Materials and Methods

### 4.1. Patients

The study was performed in accordance with the Declaration of Helsinki and approved by the Ethical Review Board of the Careggi University Hospital (reference n. 10709 approved on 27 April 2017). The study included a total of 45 infertile women (age 35.0 ± 3.3 years) with a body mass index (BMI) of 22.7 ± 4.0 kg/m^2^ and a duration of infertility of >2 years (3.2 ± 1.9 years) undergoing IVF, who were recruited from April 2017 to September 2017. Out of these, 19 women with endometriosis (stages III and IV) were included as the study group. The control group included 45 women (infertility due to tubal factor or male factor). The basic characteristics of the study population are summarized in Table 3.

From each patient, peripheral blood samples were obtained at the time of egg retrieval, FF from the mature follicles of each ovary was centrifuged, and aliquots were frozen at −80 °C until analysis. After centrifugation (1500× *g* for 15 min at 4 °C), aliquots of plasma were used for experiments or stored at −80 °C for further analyses. Granulosa cells were isolated from all of the aspirated FF using gradient centrifugation at oocyte retrieval, and immediately analyzed. Patients were enrolled according to the following inclusion criteria:

(i) Absence of any metabolic or endocrine system-associated diseases (such as hyperprolactinemia and thyroid dysfunction), and no history of cancer;

(ii) Serological markers for hepatitis B virus (HBV), hepatitis C virus (HCV), and human immunodeficiency virus (HIV) all negative;

Patients gave their written informed consent, and did not receive any monetary compensation for participating in the study. Basal follicle stimulating hormone (FSH), luteinizing hormone (LH), and estradiol (E2) levels were tested on cycle day 3 of a spontaneous menstrual cycle prior to ovarian stimulation.

All of the recruited patients received the antagonist protocol for ovarian stimulation. The ovarian stimulation began with 125–225 IU of recombinant FSH (Gonal-F^®^; Merck Serono; Darmstadt, Germany) from day 2 of the menstrual cycle, and the GnRH antagonist (Cetrotide; Merck Serono, Darmstadt, Germany) was introduced according to a multiple-dose protocol (0.25 mg/day) when a leading follicle of 14 mm and/ or estradiol concentrations of 400 pg/mL were reached. Triggering was performed when at least three follicles >17 mm were present with 0.2 mg of triptorelin SC (Decapeptyl, Ipsen Pharma, Paris, France) or recombinant human chorionic gonadotrophin (HCG) (Ovitrelle^®^, Merck Serono) and oocyte retrieval was performed under sedation at the 36th hour following GnRHa.

### 4.2. FF Collection and Processing

FF aspiration was performed transvaginally using a transvaginal ultrasound probe as a guide, and a specific oocyte aspiration needle connected to a closed vacuum system was used to empty the follicles. Once separated, oocytes were placed into culture media, while FF was collected in flasks [11].

Flushing was not used in our study protocol. Follicular selection was performed according to follicular size, assuming that the representative size reflecting oocyte maturity and fecundation capacity is >18 mm, thus disregarding smaller follicles in the process. Then, only the pure follicular fluid of mature oocytes (MII stage) was analyzed. We did not use any medium, and we did not perform any washing step. The follicular fluid of immature oocytes (germinal vesicle or metaphase I) were not considered for the study. Blood contamination was evaluated by visual inspection, and samples that looked cloudy or bloodstained were discarded [47]. Only uncontaminated FF minimally stained with blood was used for analysis. Samples without oocytes or that were contaminated were discarded.

### 4.3. Total Antioxidant Capacity (TAC) Assay

The ORAC method (oxygen radical absorbance capacity), based on the inhibition of the peroxyl radical-induced oxidation initiated by thermal decomposition of azo compounds such as 2,2′-azobis(2-amidinopropane) dihydrochloride (AAPH), was performed as previously described on plasma and FF samples [48]. Briefly, a fluorescein solution (6 nM) prepared daily from a 4-μM stock in 75-mM sodium phosphate buffer (pH 7.4), was used. Trolox (250 µM final concentration) was used as a standard. 70 µL of each sample with 100 µL of fluorescein was pre-incubated for 30 min at 37 °C in each well, before rapidly adding AAPH solution (19 mM final concentration). Fluorescence was measured with excitation at 485 nm and emission was measured at 537 nm in a Fluoroskan Ascent Microplate Fluorometer (Thermo Fisher Scientific Inc. MA, USA). Results were expressed as Trolox Equivalents (µM), and then normalized for protein concentration.

### 4.4. TBARS (Thiobarbituric Acid Reactive Substances) Estimation

Plasma and FF TBARS levels were measured using a TBARS assay kit (OXI-TEK, ENZO Life Sciences, NY 11735, USA) in accordance with previous report by our group [49]. Briefly, the adduct generated by reacting malondialdehyde with thiobarbituric acid after 1 h at 95 °C was measured spectrofluorimetrically, with excitation at 530 nm and emission at 550 nm. TBARS were expressed in terms of malondialdehyde equivalent (nmol/mL).

### 4.5. ROS Assessment by Flow Cytometry Analysis

After collection, 100 μL of EDTA-anticoagulated blood samples or FF samples was resuspended in 2 mL of BD FACS Lysing Solution (Becton Dickinson Biosciences, San Jose, CA, USA), gently mixed, and incubated at room temperature in the dark for 10 min, as previously reported [50]. Next, cells were centrifuged, the supernatant was discarded, and cells were washed twice in PBS. To determine the level of intracellular ROS generation, cells were incubated with H_2_DCF-DA (2.5 μM) (Invitrogen, Carlsbad, CA, USA) in RPMI medium without serum and phenol red for 15 min at 37 °C. H_2_DCFDA is a chemically reduced form of fluorescein that is used as a ROS indicator in cells. The 2′,7′-di-chlorofluorescein (DCF) fluorescent probe is particularly sensitive to hydrogen peroxide, peroxynitrite, and hydroxyl radicals. Superoxide anions also can contribute to H_2_DCFDA oxidation, albeit to a lesser degree. Although other more specialized ROS probes have been—and continue to be—developed, H_2_DCFDA remains the most versatile indicator of cellular oxidative stress, and represents the gold standard for ROS measurement [51]. After labeling, cells were washed and resuspended in PBS and analyzed immediately using a FACSCanto flow cytometer (Becton-Dickinson, San Jose, CA, USA). The sample flow rate was adjusted to about 1000 cells/s. For a single analysis, the fluorescence properties of 20,000 cells were collected. The respective gates were defined using the distinctive forward-scatter and side-scatter properties of the individual cell populations. Moreover, the viability of the cells was controlled by flow cytometry with propidium iodide staining, and was found to exceed 95%. Data was analyzed using BD FACSDiva software (Becton-Dickinson, San Jose, CA, USA) [52].

### 4.6. ORAC/TBARS Ratio as an Index of Oxidative Stress

The ORAC/TBARS ratio was calculated in the plasma and FF of infertile and healthy subjects in order to obtain a measure of oxidative stress.

### 4.7. Measurements of Outcomes: Metaphase II

In the intracytoplasmic sperm injection (ICSI) procedure, in order to determine the rate of metaphase II oocytes, oocytes retrieved at 35 h post-HCG administration were exposed briefly to 80 IU/mL hyaluronidase (Origio), followed by the denudation of the surrounding cumulus cells by stepwise mechanical stripping. Oocytes were then examined under an inverted microscope at a magnification of 200× to assess the maturity stage (germinal vesicle, metaphase I, or metaphase II), and only those that have extruded the first polar body were selected for ICSI. During in vitro fertilization and embryo transfer (IVF-ET), the oocytes are surrounded by cumulus and corona cells at the time of insemination so that their maturity cannot easily be evaluated. The maturity stage assessment is performed 16–20 h after insemination, once the corona radiata has been removed using denuding pipettes.

### 4.8. Measurements of Outcomes: Fertilization Rate (FR)

The fertilization rate (FR) is the percentage of correctly fertilized oocytes (zygotes with two pronuclei) per number of inseminated oocytes; fertilization was evaluated under an inverted microscope 16–20 h after ICSI or IVF.

### 4.9. Measurements of Outcomes: Ongoing Pregnancies (at 20 Weeks of Gestation)

The establishment of a pregnancy was defined by HCG levels above 50 mU/L, and a gestational sac with a heart beat.

### 4.10. Statistical Analysis

All of the experiments were performed in triplicate. For descriptive aspects, owing to the symmetry of the distribution, data were summarized as mean ± SD. Statistical analysis was performed using the SPSS (Statistical Package for Social Sciences; Chicago, IL, USA) software for Windows (version 20.0). The nonparametric Mann–Whitney test for unpaired data was used for comparisons between single groups. Correlation analyses were measured using the Spearman correlation test. Partial correlation analyses adjusted for endometriosis, age, smoking habits, BMI, IVF-ET/ICSI, FSH, LH, estradiol, AMH and duration of infertility were also performed. *p* < 0.05 was accepted as statistically significant.

## Figures and Tables

**Table 1:** Partial correlation coefficients between the investigated oxidative stress-related parameters after controlling for endometriosis, age, smoking habits, body mass index (BMI), in vitro fertilization-embryo transfer (IVF-ET)/intracytoplasmic sperm injection (ICSI), follicle stimulating hormone (FSH), luteinizing hormone (LH), estradiol (E2), Anti-Müllerian Hormone (AMH) and duration of infertility.

Redox Parameters	Plasma ORAC	Plasma TBARS	Lymphocyte ROS	Monocyte ROS	Granulocyte ROS	FF ORAC	FF TBARS	Granulosa Cell ROS
**Plasma Redox Index** ** (RI)**	*r* = 0.838	*r* = −0.905	*r* = −0.640	*r* = −0.669	*r* = −0.724	*r* = 0.083	*r* = −0.748	*r* = −0.400
	*p* < 0.001	*p* < 0.001	*p* < 0.001	*p* < 0.001	*p* < 0.001	*p* = 0.639	*p* < 0.001	*p* = 0.019
**FF Redox Index (RI)**	*r* = 0.474	*r* = −0.506	*r* = −0.431	*r* = −0.520	*r* = −0.489	*r* = 0.761	*r* = −0.520	*r* = −0.528
	*p* = 0.005	*p* = 0.002	*p* = 0.011	*p* = 0.002	*p* = 0.003	*p* < 0.001	*p* = 0.002	*p* = 0.001
**Lymphocyte ROS**	*r* = −0.618	*r* = 0.581	-	*r* = 0.925	*r* = 0.914	*r* = −0.150	*r* = 0.664	*r* = 0.645
	*p* < 0.001	*p* < 0.001	-	*p* < 0.001	*p* < 0.001	*p* = 0.397	*p* < 0.001	*p* < 0.001
**Monocyte ROS**	*r* = −0.695	*r* = 0.621	*r* = 0.925	-	*r* = 0.937	*r* = −0.211	*r* = 0.761	*r* = 0.619
	*p* < 0.001	*p* < 0.001	*p* < 0.001	-	*p* < 0.001	*p* = 0.231	*p* < 0.001	*p* < 0.001
**Granulocyte ROS**	*r* = −0.749	*r* = 0.645	*r* = 0.914	*r* = 0.937	-	*r* = −0.134	*r* = 0.759	*r* = 0.680
	*p* < 0.001	*p* < 0.001	*p* < 0.001	*p* < 0.001	-	*p* = 0.451	*p* < 0.001	*p* < 0.001
**FF ORAC**	*r* = 0.124	*r* = −0.238	*r* = −0.150	*r* = −0.211	*r* = −0.134	-	*r* = 0.027	*r* = −0.159
	*p* = 0.485	*p* = 0.175	*p* = 0.397	*p* = 0.231	*p* = 0.451	-	*p* = 0.880	*p* = 0.368
**FF TBARS**	*r* = −0.728	*r* = 0.682	*r* = 0.664	*r* = 0.761	*r* = 0.759	*r* = 0.027	-	*r* = 0.557
	*p* < 0.001	*p* < 0.001	*p* < 0.001	*p* < 0.001	*p* < 0.001	*p* = 0.880	-	*p* = 0.001
**Granulosa cell ROS**	*r* = −0.529	*r* = 0.394	*r* = 0.645	*r* = 0.619	*r* = 0.680	*r* = −0.159	*r* = 0.557	-
	*p* = 0.001	*p* = 0.021	*p* < 0.001	*p* < 0.001	*p* < 0.001	*p* = 0.368	*p* = 0.001	-

ORAC: Oxygen Radical Absorbance Capacity; ROS: Reactive Oxygen Species; TBARS: Thiobarbituric Acid-Reactive Substances.

**Table 2:** Partial correlation coefficients between the investigated redox parameters and in vitro fertilization (IVF) outcome after controlling for endometriosis, age, smoking habits, BMI, IVF-ET/ICSI, FSH, LH, estradiol, AMH, and duration of infertility. FF: follicular fluid.

Redox Parameters	Metaphase II (%) (*n* = 45)	Fertilization Rate (%) (*n* = 45)	Ongoing Pregnancies (*n* = 12)
Plasma TBARS	*r* = −0.540; *p* = 0.001	*r* = −0.685; *p* < 0.001	*r* = −0.471; *p* = 0.005
Plasma ORAC	*r* = 0.524; *p* = 0.001	*r* = 0.469; *p* = 0.005	*r* = 0.504; *p* = 0.002
Plasma Redox Index (RI)	*r* = 0.463; *p* = 0.006	*r* = 0.520; *p* = 0.002	*r* = 0.549; *p* = 0.001
Lymphocyte ROS	*r* = −0.542; *p* = 0.001	*r* = −0.312; *p* = 0.072	*r* = −0.384; *p* = 0.025
Monocyte ROS	*r* = −0.494; *p* = 0.003	*r* = −0.367; *p* = 0.033	*r* = −0.433; *p* = 0.010
Granulocyte ROS	*r* = −0.572; *p* < 0.001	*r* = −0.333; *p* = 0.054	*r* = −0.465; *p* = 0.006
FF TBARS	*r* = −0.451; *p* = 0.007	*r* = −0.476; *p* = 0.004	*r* = −0.351; *p* = 0.042
FF ORAC	*r* = −0.212; *p* = 0.229	*r* = 0.316; *p* = 0.069	*r* = 0.404; *p* = 0.018
FF Redox Index (RI)	*r* = 0.459; *p* = 0.006	*r* = 0.413; *p* = 0.015	*r* = 0.518; *p* = 0.002
Granulosa cell ROS	*r* = −0.654; *p* < 0.001	*r* = −0.268; *p* = 0.126	*r* = −0.357; *p* = 0.038
Metaphase II (%)	-	*r* = 0.378; *p* = 0.027	*r* = 0.452; *p* = 0.007
Fertilization Rate (%)	*r* = 0.378; *p* = 0.027	-	*r* = 0.368; *p* = 0.032
Ongoing pregnancies	*r* = 0.452; *p* = 0.007	*r* = 0.368; *p* = 0.032	-

**Table 3:** Clinical characteristics of 45 infertile patients and 45 age-matched controls.

Parameters	PATIENTS (*n* = 45)	CONTROLS (*n* = 45)	
	Mean ± SD	Mean ± SD	*p*-value
Age (years)	35.0 ± 3.3	34.5 ± 4.5	not significant
BMI (Kg/m^2^)	22.8 ± 4.1	21.0 ± 1.5	0.0069
Duration of infertility (years)	3.2 ± 2.0	1.8 ± 0.8	<0.0001
FSH (mIU/mL)	8.3 ± 3.8	6.0 ± 2.2	0.0007
LH (mIU/mL)	5.9 ± 3.1	4.4 ± 2.1	0.0086
Estradiol (pg/mL)	57.1 ± 24.8	48.5 ± 3.1	0.0233
AMH (ng/mL)	2.4 ± 2.0	4.2 ± 3.6	0.0043
Smoking habits (%)	26.7	28.0	not significant
GnRH Antagonist (%)	100	100	not significant
